# Effects of Running-Specific Strength Training, Endurance Training, and Concurrent Training on Recreational Endurance Athletes’ Performance and Selected Anthropometric Parameters

**DOI:** 10.3390/ijerph191710773

**Published:** 2022-08-29

**Authors:** Pablo Prieto-González, Jaromir Sedlacek

**Affiliations:** 1 Health and Physical Education Department, Prince Sultan University, Riyadh 11586, Saudi Arabia; 2 Department of Sport Kinanthropology, Faculty of Sports, University of Prešov, 080 01 Prešov, Slovakia

**Keywords:** concurrent training, endurance training, running-specific strength training, periodization, recreational runner

## Abstract

Objective: The present study aimed to verify the effects of running-specific strength training alone, endurance training alone, and concurrent training on recreational endurance athletes’ performance and selected anthropometric parameters. Method: Thirty male recreational endurance runners were randomly assigned using a blocking technique to either a running-specific strength training group (RSSTG), an endurance training group (ETG), or a concurrent training group (CTG). RSSTG performed three strength-training sessions per week orientated to running, ETG underwent three endurance sessions per week, and CTG underwent a 3-day-per-week concurrent training program performed on non-consecutive days, alternating the strength and endurance training sessions applied to RSSTG and ETG. The training protocol lasted 12 weeks and was designed using the ATR (Accumulation, Transmutation, Realization) block periodization system. The following assessments were conducted before and after the training protocol: body mass (BM), body mass index (BMI), body fat percentage (BFP), lean mass (LM), countermovement jump (CMJ), 1RM (one-repetition maximum) squat, running economy at 12 and 14 km/h (RE12 and RE14), maximum oxygen consumption (VO_2_max), and anaerobic threshold (AnT). Results: RSSTG significantly improved the results in CMJ, 1RM squat, RE12, and RE14. ETG significantly improved in RE12, RE14, VO_2_max, and AnT. Finally, CTG, obtained significant improvements in BFP, LM, CMJ, 1RM squat, RE12, RE14, VO_2_max, and AnT. RSSTG obtained improvements significantly higher than ETG in CMJ, 1RM squat, and RE14. ETG results were significantly better than those attained by RSSTG in AnT. Moreover, CTG marks were significantly higher than those obtained by ETG in CMJ and RE14. Conclusion: Performing a 12-week concurrent training program integrated into the ATR periodization system effectively improves body composition and performance variables that can be obtained with exclusive running-specific strength and endurance training in recreational runners aged 30 to 40. Running-specific strength training enhances maximum and explosive strength and RE, whereas exclusive endurance training improves VO_2_max, AnT, and RE. Performing concurrent training on non-consecutive days effectively prevents the strength and endurance adaptations attained with single-mode exercise from being attenuated. The ATR periodization system is useful in improving recreational endurance athletes’ performance parameters, especially when performing concurrent training programs.

## 1. Introduction

Several sports require an adequate levels of strength and endurance to perform at optimum level in competitive events. However, successfully combining endurance and strength training represents the highest complexity in exercise prescription [1]. It has often been speculated that concurrent training does not generate the same adaptations as single-mode exercise [1]. Even so, the possible mechanisms whereby concurrent training of both fitness components can attenuate strength and endurance adaptations remain unclear [2].

In endurance sports, it has traditionally been thought that cardiovascular capacity is the main limiting factor in sports performance [3]. Therefore, maximum oxygen consumption (VO_2_max) and anaerobic threshold (AnT) have been considered the best indicators to predict athletes’ performance [4]. Nevertheless, in reality, endurance athletes with similar VO_2_max may perform differently in sports competitions. Hence, VO_2_max could not be the best indicator to predict their racing performance. Nowadays, running economy (RE) and the evaluations that imply assessing the muscular power exerted or the speed reached by an athlete during the VO_2_max are considered better sports performance indicators [5,6,7]. In this way, specific scientific evidence indicates that combining endurance and strength training generates additional benefits in terms of athletic performance improvement and injury prevention [8]. These improvements could be related to the following mechanisms [8,9,10,11,12,13]:

(a) Musculotendinous factors: Improved muscle-tendinous stiffness and stretch-shortening cycle properties, conversion of fast-twitch type IIx into more fatigue-resistant type IIa fibers, and delayed activation of less-efficient type II fibers.
(b) Neuromuscular factors: Improved neuromuscular function and efficiency, improved intramuscular coordination, motor unit recruitment, and firing frequency.
(c) Physical fitness components: Improved levels of strength. This allows athletes to apply a lower relative percentage of force, which reduces the contribution of the anaerobic energy system and results in reduced fatigue, maintenance of the required application of strength over a longer period, or appliance of more strength per unit of time. Additionally, peak velocity, speed, maximum aerobic speed, and anaerobic capacity are enhanced.

All these enhancements would result in an improved RE.

Nevertheless, there are arguments against using concurrent training programs in endurance runners. Vikmoen et al. (2016) and Berryman et al. (2018) [14,15] state that sports requiring high strength levels are opposite in nature to endurance sports in terms of energy metabolism and effort duration. Therefore, developing both fitness components simultaneously would result in potential combative adaptations [14,15,16]. Actually, the main adaptations produced by endurance and strength training are not only different but also opposed: Endurance training adaptations include oxidative enzyme activity increment, mitochondrial and capillary density increment, maintenance or reduction of fiber size, and possibly also fiber type transformations (Type II into I), modifying the model of recruitment and reducing the muscle contractile capacity [17]. In contrast, strength training is associated with reduced capillary density, oxidative enzymes, and mitochondrial density, reducing the oxidative muscle capacity [10,15].

In this regard, some studies have revealed a certain degree of incompatibility between endurance and strength training [9,18,19]. Thus, maximum voluntary contraction, rate of force development and some adaptations such as maximal strength and VO_2_max can be attenuated [20]. Similarly, time to exhaustion and mitochondrial density can be reduced [14]. This phenomenon is known as the interference effect or concurrent training effect [11], and the potential interferences can be chronic and acute [2,11]. 

The reasons why the interference effect occurs are [1,2,11] the mechanism of muscle fiber recruitment used, the transformation of fast twitch muscle fibers into slow twitch muscle fibers, and the functioning of the endocrine system. From the molecular point of view, the simultaneous activation of cellular biomarkers that elicit optimal anabolic and endurance responses is also not possible [11]. Moreover, muscle hypertrophy implies the cross-sectional area increment of muscle fibers, which may increase the distance between the capillaries inside the muscle and could negatively impact performance. Even so, in untrained individuals, there is an increase in (or at least a maintenance of) the number of capillaries surrounding each muscle fiber, and the capillaries per fiber area do not experience modifications. However, since performing concurrent strength and endurance training can mitigate the hypertrophic response, and endurance-trained athletes have a greater number of capillaries than untrained athletes, these findings may not apply to experienced endurance athletes [14].

In line with the existence of arguments for and against the use of concurrent training, some studies have found improvements in sports performance after using strength training in endurance athletes [6,11], whereas no beneficial effect was observed in other research [14,21]. The discrepancies between studies may be due to the development of different types of strength, external variables not directly related to the intervention, the use of different training methods [12,22], or the application of training methods lacking scientific rigor [23]. 

As a result, it is essential to continue searching for better strategies to improve athletic performance by implementing strength training sessions on endurance training programs and preventing the interference between both fitness components [11,16]. Future studies should focus on minimizing the interference effect when concurrent training is applied, and training load organization, type, order, and optimal application are crucial to attain this goal. Further research is also needed to understand better the relationship between strength training, anaerobic metabolism, and endurance sports performance. New studies must be longer in duration, since the greatest increases in RE occur after implementing training protocols of more than 24 sessions, and most of the existing studies are shorter [15]. Moreover, valid strength assessments through a range of different velocities must be used, implementing adequate strength training programs over a long-term intervention period, and using multi-joint strength, explosive-strength, or reactive-strength exercises due to their superior functionally [6].

Future studies must also integrate the intervention design into a suitable periodization system and apply sports training principles to synchronize all training contents [20]. In this respect, to the best of our knowledge, at present, only one investigation has been conducted to verify the effect of strength training on endurance runners by using a periodization system [21]. Additionally, athletes’ training programs must be adapted to their personal needs and abilities. Thus, according to the law of diminishing returns, individuals’ ability to attain specific adaptations will depend on their training level. Less-trained subjects are likely to obtain greater adaptations since they have a greater adaptation reserve. In contrast, well-trained individuals need more demanding training stimuli to attain improvements throughout the training process [22].

Importantly, most existing studies related to concurrent training have focused on verifying the compatibility of simultaneous strength and endurance training. However, very few of them have examined the influence of strength, and particularly of running-specific strength training, on endurance performance [24]. Most studies examined the interference of endurance on maximal strength and hypertrophy, but not the opposite [11]. In this context, the utility of strength training remains to be clarified for endurance athletes, and research findings are often inconclusive [22]. For this reason, it is necessary to continue investigating this topic [17]. In fact, sports scientists have studied new ways to enhance biomechanics, technique, energy production, sports equipment, injury prevention, and recovery. However, concurrent training and the effect of strength training in endurance athletes is a very complex phenomenon that requires new research.

## 2. Objective

The main objective of the present study was to verify the effect of running-specific strength training, endurance training alone, and concurrent training on physiological performance and selected anthropometric variables in recreational runners. We also aimed to ascertain if 3-day-per-week concurrent training performed on non-consecutive days attenuates the strength, endurance, and anthropometric adaptations compared to strength and endurance training in isolation.

## 3. Materials and Methods

A quasi-experimental randomized study was conducted.

### 3.1. Subjects

Thirty male recreational endurance runners participated in the present research. They were assigned into three different groups: a running-specific strength training group (RSSTG) [age: 34.7 (2.36); height: 1.77 (0.04); weight: 67.05 (5.37); BMI: 21.27 (1.87)], an endurance training group (ETG) [age: 35.1 (2.77); height: 1.78 (0.05); weight: 65.80 (4.38); BMI: 20.68 (1.31)], or a concurrent training group (CTG) [age: 34.3 (2.37); height: 1.76 (0.03); weight: 66.96 (4.48); BMI: 21.48 (1.03)]. RSSTG performed thrice-per-week strength training program orientated to running on non-consecutive days. ETG performed thrice-per-week endurance training on non-consecutive days. Finally, CTG underwent 3-days-per-week concurrent training performed on non-consecutive days. CTG alternated the running-specific strength training sessions performed by RSSTG and the endurance training sessions underwent by ETG. The inclusion criteria were (a) be an active recreational runner; (b) able to run one km in less than 4:30 min; (c) have practiced running at a recreational level for at least the past five years before participating in the current research; (d) perform endurance training regularly, with a weekly frequency of not less than three times a week, but no more than five times a week; (e) have not performed endurance or strength systematic training for the last year leading up to the research’s commencement; (f) non-smoker; (g) do not use nutritional supplementation; (h) do not suffer from chronic diseases or ongoing injuries; (i) aged between 30 and 40 years old.

Participants were asked not to modify their dietary habits or lifestyle during the intervention process, and attendance was recorded. Subjects were required to attend at least 90% of the training sessions to be included in the study. Similarly, participants were informed that they could voluntarily withdraw from the study at any time. The research was conducted according to the ethical principles of the Declaration of Helsinki. It was approved by the Ethics Commission of Prešov University (Prešov, Slovakia) (ethical clearance number: 2/2021). Similarly, all subjects who participated in this study were required to submit written informed consent. Previously, they received a verbal and written explanation about the experimental design and the potential risks and benefits of participating in this research.

### 3.2. Randomization

A blocking design was used to avoid any possible bias when the subjects were allocated into three different experimental groups and to ensure the trial’s proper randomization. The blocking factor was the VO_2_max value obtained through the incremental load test. According to the results obtained in this test, participants were allocated to one of the ten blocks created. Athletes who obtained the best three marks were assigned to block one. Athletes who obtained the fourth, fifth, and sixth-best marks were assigned to block two, and so on. Afterward, each block’s three members were randomly assigned to one of the three different experimental groups. Therefore, there was one member of each block in each experimental group. 

### 3.3. Training Protocol

The intervention lasted 12 weeks, and study participants performed three sessions per week on non-consecutive days. The training protocol was designed using the ATR block periodization system. The training intervention was divided into three mesocycles: accumulation (first six weeks), transmutation (from week seven to week 10), and realization (weeks 11 and 12). The duration of the training protocol was the minimum necessary to attain the planned adaptations in accordance with the abilities developed in each mesocycle, the sports discipline, and the characteristics of the athletes. The training methods applied to RSSTG are shown in Table 1, and the training methods that ETG underwent are shown in Table 2. 

Likewise, CTG underwent 3-day-per-week concurrent training performed on non-consecutive days, alternating the strength and endurance sessions carried out by RSSTG and ETG. All training sessions were supervised by the same researcher: a One Physical Education Bachelor’s Degree holder, expert in sports training, and with more than 20 years of working experience in the sports field. Likewise, all training sessions were conducted in the same fitness center to minimize external variables’ influence avoid compromising the results’ validity and.

### 3.4. Assessments

Physiological and selected anthropometric parameters were measured before (pre-test) and after (post-test) applying the 12-week training protocol. The assessments were conducted after a rest period of 48 h, between 5:00 p.m. and 7:00 p.m. The study participants were asked to refrain from ingesting food or beverages three hours before testing. To avoid the learning effect, a theoretical–practical training session was conducted one week before the pre-test. The main researcher explained the testing protocols in detail. Thereupon, the participants practiced the proper technique of execution of all tests to verify the correct functioning of the equipment and procedures used in the assessment. The following two-phase warm-up was performed before conducting the physical and physiological tests: General warm-up: 10 min running at 60% of their theoretical maximum heart rate plus five minutes of joint mobilization exercises. Specific warm-up: 2 × 10 vertical jumps, three sets of squats (10 reps at 50% of their estimated 1RM, five reps at their estimated 70% 1RM, and three reps at their estimated 80% 1RM), and one set of 20 m acceleration. The theoretical 1RM was estimated based on the information collected during the theoretical–practical training session conducted one week before the pre-test. In addition, the subject’s BM, age, and strength training experience were also taken into account.

Assessments included in the pre-test and post-test are detailed below:

- Body mass (BM) and body mass index (BMI): Both were measured using a Seca digital column scale, model 769 (Hamburg, Germany). Height was measured to the nearest 0.1 cm and body mass to the nearest 0.1 kg. Body mass and body mass index were assessed by the same investigator, and the subjects were in bare feet.
- Body fat percentage (BFP): BFP was obtained through the following equation [25]: BFP = [(Σ of abdominal, subscapular, triceps, suprailiac, abdominal, thigh, calf)0.143] + 4.56. The plicometer used to measure the fat folds was a Harpenden Skinfold Caliper, model FG1056 (Sussex, UK).
- Lean mass (LM): LM was calculated by using the following formula: LM = Body Mass (kg) − (Fat Mass (Kg) × BFP).
- Countermovement jump (CMJ): CMJ was used to assess jumping ability and lower body power due to its high reliability and validity [26]. The test was performed in one indoor gymnasium on a dry and non-slippery surface. The device used was an Optojump-next (Bolzano, Italy) connected to one laptop via USB, and the Microgate software (Optojump software, version 3.01.0001) was also utilized. Participants were not allowed to use the arm swing. Instead, they were required to keep their hands on their hips while performing the test. The test started with a quick countermovement action. When the knees were bent 90°, athletes initiated the take-off and flight. During the flight, they had to maintain their hips, knees, and ankles extended. Participants were also required to jump vertically. Movements forward, backward, or sideways were not allowed [27]. Each subject had two attempts, with three minutes rest between each jump.
- One-repetition maximum (1RM) squat: This test was used to measure lower body maximum strength due to its validity, reliability, and applicability [28,29]. To conduct the test properly, participants kept their trunks naturally upright. The bar was grasped firmly with both hands, and it was also supported on the shoulders. The test started with knees bent at 90° until the performer’s thighs were parallel with the ground. Then, subjects regained the upright position, with the legs fully extended. The number of attempts required to determine the 1RM of each subject was between two and four. Only the attempts correctly performed were registered. The resting time between attempts was three minutes.
- Incremental load test: This laboratory test was used to assess the Anaerobic Threshold (AnT), maximal oxygen consumption (VO_2_max), and running economy (RE). The following protocols were implemented:
  - Protocol I: The objective was to determine the RE by using one treadmill (Cybex 625T, Rosemont, IL, USA), one metabolic gas analyzer (Cosmed Srl, Albano Laziale, Rome, Italy), and one heart rate monitor (Polar H9 BLE Kempele, Finland). The protocol comprised an 8 min submaximal test consisting of two, four-minute stages. That is to say, each stage was four minutes in length to allow for oxygen consumption and steady-state heart rate. The first stage was performed at 12 km/h, and the second at 14 km/h. These two speeds are within the intensity range established by Barnes and Kilding (2015) to measure RE in recreational athletes [30]. Then, to determine the RE values, the VO_2_ mean value of the last minute of each stage was recorded [31].
  - Protocol II: This test was used to estimate the VO_2_max and AnT. The treadmill’s initial velocity was set at 2 km/h slower than the subjects’ estimated 4 mmol when the test started. Then, the speed was increased by 0.5 km/h every 30 s until exhaustion [32].

### 3.5. Statistical Analysis

Data is presented using the format mean SD (standard deviation). The Shapiro–Wilk test was used to contrast the normality of the variables and Levene’s test to verify the homogeneity of variances. Sphericity assumptions were assessed using Mauchly’s test. When those sphericity assumptions were violated, the Greenhouse Gessier correction was applied. To determine the concordance between the pre-test and post-test measurements, the interclass correlation coefficient (ICC) was calculated for all the assessed anthropometric and performance parameters. ICC values were interpreted as follows: ICC ≤ 0.49, poor; 0.50 ≤ ICC < 0.75, moderate; 0.75 ≤ ICC < 0.9, good; ICC ≥ 0.9, excellent [33]. To verify whether there were differences between groups in the baseline, a one-way ANOVA (analysis of variance) test was conducted. To assess the training effects between groups (ETG vs. RSSTG vs. CTG) and within groups (pre-test vs. post-test) on the anthropometric and performance variables, a two-way repeated-measure ANOVA was performed. When statistically significant *p* values were found (group by time interaction effect or significant main effects of time or group), a post hoc pairwise comparison was conducted with Bonferroni correction to identify those differences. The effect size was calculated using Cohen’s d. Values of d < 0.2, d = 0.2, d = 0.5, and d = 0.8 were considered as trivial, small, medium, and large effect sizes, respectively [34]. The level of significance established was *p* < 0.05. The statistical analysis of the data was performed using the program IBM SPSS V.26^®^ computing (IBM Corp., Armonk, NY, USA). 

## 4. Results

Once the normality and homoscedasticity of the data were verified, the one-way ANOVA confirmed the absence of significant differences between the three experimental groups at the baseline for all the anthropometric and performance variables. Likewise, the ICC values obtained between the pre-test and the post-test in all assessed parameters were higher than 0.9 for the three groups, which indicates an excellent reliability. Then, the two-way repeated-measure ANOVA showed that there was no interaction effect, main effect of time, or group for BM and BMI (see Table 3). Furthermore, the two-way repeated-measure ANOVA revealed the existence of a group-by-time interaction effect for CMJ, 1RM squat, RE12, RE14, VO_2_max, and AnT. A main effect of time was observed for the BFP, LM, CMJ, 1RM squat, RE12, RE14, VO_2_max, and AnT. Finally, a main effect of group was found for CMJ and RE14 (see Table 3).

Subsequently, the Bonferroni post hoc comparison showed that ST obtained improvements significantly higher than ET enhancements in the following variables: CMJ (*p* = 0.003; CI95 = 1.81–7.51), 1RM squat (*p* = 0.035; CI95 = 1.33–9.86), and RE14 (*p* = 0.046; CI95 = 46.69–5036.38). ET results were significantly better than those obtained by ST in AnT (*p* = 0.04; CI95 = 0.04–1.95). Finally, the improvements obtained by CT were significantly higher than those attained by ET in CMJ (*p* = 0.002; CI95 = 1.21–4.26), and RE14 (*p* = 0.046; IC95 = 47.47–5035.51).

As for the within-subject comparisons (see Table 4), RSSTG significantly improved between the pre- and post-tests in CMJ (*p* < 0.001; IC95 = 3.42–4.13), 1RM squat (*p* < 0.001; IC = 5.66–8.73), RE12 (*p* < 0.001; 5.66–8.73) and RE14 (*p* = 0.007; F: 0.23–1.11). The effect size of these improvements was small in the case of RE12 and RE14, and large for CMJ and 1RM squat. ETG significantly improved between the pre- and post-tests in the following parameters: RE12 (*p* < 0.001; IC95 = 1.93–2.59), RE14 (*p* = 0.015; F = 620.65–4464.61), VO_2_max (*p* < 0.001; CI95 = 0.51–0.77), and AnT (*p* < 0.001; CI95 = 262–738). The effect size of these improvements was small in the case of RE14, medium for VO_2_max, and large for RE12 and AnT. Additionally, CTG significantly improved its results between the pre- and the post-tests in the following variables: BFP (*p* < 0.001; CI95 = 0.354–0.590), LM (*p* = 0.035; CI95 = 0.5–1.12), CMJ (*p* < 0.001; CI95 = 1.72–2.47), 1RM squat (*p* < 0.001; CI95 = 3.10–4.10), RE12 (*p* = 0.035; CI95 = 1.62–2.56), RE14 (*p* < 0.001; CI95 = 0.80–1.84), VO_2_max (*p* < 0.001; CI95 = 1.57–3.02), and AnT (*p* < 0.001; 0.84–1.15). The effect size of these improvements was small in the case of RE14, medium for BFP and RE12, and large for the CMJ, 1RM squat, and AnT variables.

## 5. Discussion

### 5.1. Anthropometric Parameters

None of the three experimental groups presented modifications to their BM in the present study. Furthermore, the effect sizes of the modifications produced in this parameter between the baseline and the post-test were trivial in all three cases. These results are consistent with previous recent studies [12,32,35]. Thus, on the one hand, it can be expected that the exclusive practice of endurance training may promote muscular catabolism and increase mitochondrial density and activity. Therefore, these adaptations could reduce body mass and body fat percentage [36]. On the other hand, strength training can potentially increase lean tissue by increasing the release of anabolic hormones such as testosterone and growth hormone [37,38]. However, in the present study, we understand that the absence of significant modifications in BM may be due to the following reasons: Firstly, the duration of the intervention period (12 weeks) could not be long enough to generate significant variations in this parameter. Secondly, in RSSTG and CTG, the potential or expected decrease in BM of the participants derived from the slight reduction in fat mass has been hindered by the slight increase in LM. Thus, the net result would be a non-significant alteration of athletes’ BM. Likewise, since the study participants were recreational athletes who perform endurance training sessions regularly, to be able to attain significant decreases in their body weight, it is plausible that they require not only more extended intervention periods, but also significant increases in their weekly training frequency, volume, density, and intensity.

Regarding ETG, the absence of a significant weight reduction might be related to the participants’ regular practice of endurance training. Therefore, this group may need more intense or prolonged workouts to significantly decreases BM. Importantly, there was no increase in lean tissue in ETG but a slight reduction. This means that the slight decrease in lean tissue of the participants included in this group, together with their slight reduction in BFP, was of such small magnitude that it did not cause a significant reduction in BM. This is in line with the trivial effect sizes observed in ETG in its reduction of BFP and LM.

As for the BMI, there were no significant variations in any of the three experimental groups. In the case of RSSTG and CTG, a certain increase in their BMI might be expected due to the strength training practice and its potential anabolic effect. However, the effect size of the BMI increase in both groups was trivial. These results are somewhat surprising, since none of the study participants had previous experience in strength training. Moreover, it must be taken into account that, although the objective of the strength training protocol used in the present research was not designed to produce muscle hypertrophy (see Table 1), strength training is likely to generate certain amount of hypertrophy, particularly in subjects without previous strength training experience [39]. These results are more surprising in the case of RSSTG, since they did not perform endurance training. Thus, the trivial increase in RSSTG suggests that for strength training to generate significant increases in BMI, it is necessary to use training methods specifically aimed at achieving this goal and, probably, a longer intervention period. In ETG, as expected, the BMI did not increase but decreased. However, the reduction was trivial. We understand this slight decrease is in line with the training principle of specificity since endurance training is more likely to generate reductions in BM and BMI rather than increases [36].

As far as the BFP is concerned, significant reductions were only observed in CTG. These results coincide with the study carried out by Eklund et al. (2016) [40]. In contrast, the BFP remained unaffected in some studies after applying a concurrent training protocol [12,32]. Furthermore, Blagrove et al. (2018c) conducted a systematic review to analyze the effects of adding strength training to the endurance training programs of medium- and long-distance athletes. They observed that BFP is commonly unaffected [41]. In the present study, we understand that only CTG significantly improved its BFP due to the strength and endurance training combination. This could be because strength training can increase basal metabolism [39,42], and endurance training may produce a significant caloric expenditure [39,42,43]. Regarding ETG, despite the fact that endurance training effectively reduced BFP in some previous studies [38,44], we understand that subjects included in this group did not improve their BFP due to the absence of strength training, which implied that they could not benefit from its potential capacity to increase basal metabolism. On the contrary, we interpret that RSSTG could not significantly decrease their BFP since they did not practice endurance training and could not benefit from the significant caloric expenditure that endurance training produces.

Concerning the LM, only CTG significantly increased this parameter after undertaking the 12-week-training protocol. This increase could be related to the following reasons. First, a greater training variability was applied to this group. Second, only CTG significantly decreased BFP. Therefore, even though CTG and RSSTG reduced their LM in absolute terms at similar levels and with similar effect sizes, the increase in CTG in relative terms was higher due to its greater reduction in BFP. Third, according to Coffey and Hawley (2017) and Fyfe and Loenneke (2018), the practice of divergent exercise (i.e., strength and endurance) by untrained or recreationally active individuals induced similar increased anabolic signaling in skeletal muscle during the first weeks of training [1,22]. Not surprisingly, ETG did not increase the LM. Unlike CTG and RSSTG, this group decreased its LM, but not significantly. We consider that this could be related to the potential catabolic effect of aerobic exercise [36]. The LM results of the present study are consistent with those attained by Eklund et al. (2016) and Vikmoen et al. (2020) [40,45]. However, our results only partially coincide with those obtained by Vikmoen et al. (2017) since they observed that both concurrent strength–endurance training and running-specific strength training are useful for increasing LM [46]. Furthermore, Beattie et al. (2017) did not observe any changes in LM in competitive distance runners, probably because it is more difficult for well-trained subjects to attain adaptations due to their lower reserve of adaptation [32]. 

### 5.2. Performance Variables

As expected, ETG did not improve the results in CMJ. This confirms that endurance training does not significantly modify the marks obtained by recreational endurance athletes in CMJ. In contrast, RSSTG and CTG significantly improved their performance in CMJ. In the first case, it was expected since RSSTG only performed running-specific strength training sessions, and the subjects included in the present study had no previous strength training experience. As for CTG, it has been verified that their improvements in CMJ produced by the strength training performed were not attenuated despite the concomitant strength and endurance training. Additionally, RSSTG and CTG obtained significantly better results than ETG in CMJ. This proves that the interference effect does not occur with the weekly training frequency used in the training protocol. The results of the present study are consistent with the findings obtained by Fyfe, Bishop and Stepto (2014) [47]. These authors state that there is no evidence supporting the interference effect theory. In this regard, Coffey and Hawley (2017) add that despite chronic studies indicating that there is robust evidence supporting that endurance training attenuates strength adaptations when concurrent training protocols are applied, the underlying mechanisms of the mentioned interference are unknown. Nevertheless, some studies have verified that endurance training attenuates improvements in power, specifically in CMJ when concurrent training protocols are implemented [45,48].

As for 1RM squat, as expected, RSSTG and CTG improved their marks between pre- and post-test, probably because the study participants had no previous strength training experience. Only the marks obtained by RSSTG were significantly higher than those attained by ETG in the post-test. This could indicate that a higher frequency of strength training provides additional benefits, since the number of strength sessions performed by RSSTG was higher than the sessions performed by CTG. However, we understand that the interference effect did not occur in CTG, because no significant differences between RSSTG and CTG were observed in the post-test in 1RM squat. The present research results are consistent with those obtained by Vikmoen et al. (2016), Vikmoen et al. (2017), and Sousa et al. (2017) [14,46,49]. In all three cases, the utility of concurrent training to improve the 1RM squat was verified.

As far as RE is concerned, the three groups significantly improved this parameter at 12 and 14 km/h. The improvements obtained in RE14 by CTG and RSSTG were significantly higher than those achieved by ETG. In RSSTG, the improvements in RE could be related to the attainment of certain adaptations [9,10,32,50]: (a) improved musculotendinous stiffness of the lower extremities; (b) improved motor unit recruitment and synchronization patterns; (c) improved intermuscular coordination and neural inhibition; (d) delayed activation of less-efficient type II muscle fibers; (e) conversion of type IIx fibers into fatigue-resistant IIa fibers; (f) facilitation of the optimal application of strength throughout the entire training or competition; (g) reduction of the relative intensity that each particular cycle of effort or sports technique represents for one athlete when overcoming the same resistance; (g) improved ability to perform the same effort with lower oxygen consumption; (h) improved ability to apply the same strength with less muscle mass; (i) improved reuse of elastic energy in each stride. Therefore, attaining all of these physiological adaptations could be the reason why RSSTG obtained significant improvements over ETG in RE14.

Regarding the results obtained by ETG in RE12 and RE14, we interpreted that the improvements were the result of attaining certain adaptations [51]: (a) improved oxidative capacity, which in turn is associated with better mitochondrial functioning, and leads to a reduction in the use of the oxygen required to perform submaximal intensity efforts; (b) improved buffering capacity of the skeletal muscles and hematological system. As for CTG, we considered they simultaneously benefited from the adaptations that both strength and endurance training provide to improve RE. This circumstance would explain why CTG obtained significantly better results than ETG in RE14. Likewise, it is also understandable that the results obtained by ETG were significantly lower than those attained by RSSTG and CTG in RE14 but not in RE12, since runners show greater RE values at race pace [12]. In this regard, considering the characteristics of the athletes included in the present research and their results in AnT (see Table 3), it is understood that their competition race velocity is close to 14 km/h. The results of the present research are consistent with the studies of Beattie et al. (2017), Blagrove et al. (2018b), Giovanelli et al. (2017), and Li et al. (2019) [12,31,32,52]. In all four cases, the practice of concurrent training was effective in improving RE. Additionally, recent systematic reviews and meta-analyses confirmed the efficacy of strength training in improving RE [6,15,41]. In contrast, some studies verified that the implementation of concurrent training programs was not effective in enhancing RE [14,53].

Regarding the VO_2_max, although the trainability of this variable could be conditioned by genetic factors [54], ETG and CTG obtained significant improvements. Therefore, it can be assumed that these improvements are training-specific adaptations. Likewise, the fact that the improvements attained by ETG were not significantly higher than those achieved by CTG suggests that no interference effect has occurred. The present study results coincide with the research conducted by Patoz et al. (2021) [53]. However, in a systematic review, Blagrove et al. (2018c) verified that VO_2_max is typically unaffected after the application of concurrent training programs [41]. Therefore, the discrepancies between studies could occur because the possibility of improving VO_2_max is genetically conditioned [54]. Moreover, as expected, RSSTG did not improve the VO_2_max, probably because this group did not perform endurance training sessions. In fact, few studies found significant improvements in VO_2_max after the exclusive practice of strength training. In this regard, Ozaki et al. (2013) conducted a review study to verify the effects of strength training on increasing VO_2_max, and in only three out of the 17 studies analyzed were significant improvements in VO_2_max registered. They also ascertained that the higher the training level, the more difficult it is to improve the VO_2_max [55].

Finally, regarding AnT, both ETG and CTG improved this variable. We understand that this improvement resulted from the training methods specifically designed to enhance the AnT (see Table 2). Likewise, the results attained by ETG were significantly better than those achieved by RSSTG. However, the absence of significant differences between ETG and CTG reveals that no interference effect has occurred in CTG. Furthermore, as expected, RSSTG did not significantly improve the AnT. In this case, we consider that the absence of endurance training explains the non-achievement of significant improvements. Moreover, few studies have examined the effects on AnT. Ferrauti et al. (2010) verified the absence of significant differences in AnT between a concurrent and endurance training program in isolation [35]. Likewise, Cragnulini (2016), after conducting one review article, concluded that adding strength training to endurance athletes’ training programs does not have a negative impact on AnT [17].

### 5.3. Overall Interpretation of the Results

The improvements attained by the three experimental groups are specific exercise mode adaptations. Thus, RSSTG improved all strength parameters, ETG all the endurance parameters, and CTG strength and endurance parameters, and only the concurrent training program effectively improved body composition in 12 weeks. Additionally, the interference effect did not occur for the strength, endurance, or anthropometric variables. This suggests that the weekly frequency used in the training protocol of the present study prevents the attenuation of adaptations in concurrent training protocols with respect to single-mode strength or endurance exercise. In this regard, Pattison et al. (2020) point out that CMJ is useful for analyzing the interference effect on neuromuscular improvements when performing concurrent training programs [48], and the results obtained in the present research by RSSTG in CMJ were not significantly better than those attained by CTG.

Furthermore, based on the improvements attained by RSSTG and CTG in 1RM squat and CMJ, and also considering the large effect sizes obtained by both groups, it can be inferred that athletes without previous strength training experience can obtain significant improvements in key performance parameters due to their greater reserve of adaptation. In this sense, Fyfe and Loenneke (2018) consider that untrained individuals have a greater capacity to adapt to training stimuli than trained individuals, although their individual genetic potential could also limit the possibility of obtaining improvements [22]. For this reason, Beattie et al. (2014) indicate that, for endurance athletes with lower levels of strength, a general strength training program may be sufficient to improve their maximum strength, explosive strength, and reactive strength [6]. However, athletes with higher strength levels should perform explosive and reactive strength training programs to improve their performance [6,56].

It is also noteworthy that, based on the improvements achieved by the three experimental groups after the 12-week training program, the ATR periodization system seems to be adequate to improve the performance of recreational endurance runners, mainly when concurrent training programs are applied. However, it is also possible that intervention periods longer than 12 weeks can be required to attain significant improvements in body composition, mainly when a single-mode exercise is used. Regrettably, few studies used concurrent training protocols integrated into periodization systems such as the ATR block periodization system. In this regard, García-Manso et al. (2017) conducted one research with recreational college-age subjects, using one block periodization for nine weeks. They verified that concurrent and exclusive endurance training effectively improves sports performance. However, no significant differences were found between both training protocols [21].

Importantly, we must mention future lines of research. Despite several studies examining concurrent training programs’ effects on endurance athletes, many aspects remain unclear. This circumstance is further aggravated by the important methodological differences that exist between studies and their limitations. Thus, future studies might consider the following aspects:

(a) The use of concurrent training programs might be oriented to ensure that neuromuscular adaptations positively impact athlete’s biomechanical and performance parameters [57].
(b) It could be interesting to investigate the effect of different running volumes combined with explosive strength training [58].
(c) Since an eight-week concurrent training program could not be long enough to improve certain performance variables [35], long-term intervention periods might be applied. Li et al. (2019) propose the use of training protocols longer than 16 weeks [31], and Beattie et al. (2014) longer than six months [6].
(d) Although Berryman et al. (2018) found that the beneficial effects of strength training on endurance performance occur regardless of athletes’ level [15], some authors propose conducting studies with different populations. Low et al. (2019) recommend conducting research with women and people with different training status [59], and Li et al. (2019) with groups of senior citizens and women [31].
(e) Future training protocols might be integrated into periodization systems such as the ATR model. In this vein, Berryman et al. (2018) point out that the use of periodization strategies could help clarify the optimal moment to implement strength training activities within the annual training plan [15].
(f) Future studies might include training protocols combining different endurance training methods (i.e., fartlek, continuous training, interval training) [23].
(g) Appropriate training methods and tests to develop and assess strength levels must be used [6].
(h) The number of strength training sessions per week might be between two and three [41].
(i) The training protocols used might be designed in accordance with the sports training principles.

Finally, it is necessary to mention the study’s strengths and limitations. As for the strengths, there are two noteworthy aspects. First, as well as including one concurrent and one single-mode endurance training group, one running-specific strength training group was incorporated. This circumstance (which is not usual in studies conducted with endurance athletes) was useful to verify the adaptations that endurance runners can attain with running-specific strength training and determine the possible existence of interference effects in CTG. Second, the number of weekly training sessions applied to the three experimental groups was equated. In this regard, it should be noted that in several previous studies, the concurrent group performed two or three additional weekly strength sessions with respect to the endurance training group, which implies using a distinctive number of sessions.

As for the limitations, first, the sample size was small. A larger sample would have ensured greater representativity. Second, no time-trial test was conducted in the present research since the study participants were not specialized in any specific distance. Finally, we consider that if the intervention period had been longer, it would have been possible that the three experimental groups had obtained additional improvements, particularly in body composition. Furthermore, significant differences between groups could have occurred in more anthropometric and performance variables at the post-test.

## 6. Conclusions

A concurrent training program of 12 weeks integrated into the ATR periodization system is effective in enhancing body composition and selected sports performance parameters associated with the exclusive practice of strength training (maximum and explosive strength) and endurance training (VO_2_max and AnT), in addition to RE in recreational runners aged 30–40. The exclusive practice of running-specific strength training during the same period also using the ATR design improves maximum and explosive strength and RE, while the exclusive practice of endurance training using the ATR model improves endurance parameters (VO_2_max and AnT) and RE. Thus, concurrent training is the most time-efficient method to attain anthropometric and performance adaptations. Additionally, a concurrent training program performed on non-consecutive days did not attenuate the endurance and strength adaptations that can be attained with single-mode exercise. However, it cannot be ruled out that a higher weekly training frequency may generate interference effects.

The ATR periodization system improves the performance parameters of recreational endurance athletes, especially when performing concurrent training. Likewise, in recreational athletes without previous strength training experience, due to their greater reserve of adaptation, concomitant running-specific strength and endurance training—in addition to enhancing their body composition and relevant performance variables—produce large improvements in maximum and explosive strength and further enhance RE.

## 7. Practical Applications

- Performing exclusive strength or endurance training allows athletes only to attain specific exercise mode adaptations.
- Undertaking concurrent training programs allows athletes to obtain strength and endurance adaptations. However, to this end, separating the training sessions by at least nine hours is necessary to avoid significant interferences, or 24 h to guarantee to a greater extent that the adaptations will not be attenuated [60].
- Concurrent training programs should be integrated into a periodization model to attain greater effectiveness. In this sense, the ATR block periodization system effectively improves anthropometric and performance variables in recreational endurance athletes.
- Long-term interventions of more than 12 weeks might be used. Intervention periods of 12 weeks are insufficient to attain improvements in anthropometric parameters when single-mode exercise training is used.
- Regarding the training load and the type of strength that must be developed, it is necessary to adapt the strength training program to the athlete’s objectives, training level, and previous training experience. Thus, a general strength training program might improve sports performance in subjects without previous strength training experience. However, in subjects who did not previously develop their maximum strength, it can be leapfrogging using inappropriate workloads or developing types of strength with lower residual effect (i.e., explosive strength, muscular endurance, reactive strength). It may also reduce their adaptation reserve unnecessarily and limit future improvements. By contrast, endurance athletes with previous strength training experience should develop explosive and reactive strength to enhance their performance.

## Figures and Tables

**Table 1:** Training methodology that will be used with RSSTG.

Week	Training Parameters
1	I: 64% 1RM; S: 4; R: 14; RT: 2″; Ex: Squat, leg curl, calf raise
2	I: 69% 1RM; S: 4; R: 12; RT: 2′; Ex: Squat, leg curl, calf raise
3	I: 69% 1RM; S: 5; R: 12; RTS: 2′; Ex: Squat, leg curl, calf raise
4	I: 69%–69%–75%–75%–80% 1RM; S: 5; R: 12-12-10-10-8; RTS: 3′; Ex: Squat, leg curl, calf raise
5	I: 69%–75%–80%–85% 1RM; S: 4; R: 12-10-8-6; RTS: 3′; Ex: Squat, leg curl, calf raise
6	I: 80% 1RM; S: 4; R: 8; RTS: 3′; Ex: Squat, leg curl, calf raise
7	6S of: Squat (6R at 80% 1RM) + hurdle hops (10R) + 2′30″ running at 100% of MAS; RTS: 5’
8	6S of: Squat (5R at 82% 1RM) + hurdle hops (10R) + 2′30″ running at 100% of MAS; RTS: 5’
9	6S of: Squat (4R at 84% 1RM) + extended bounds (cover 50 m alternating legs by doing the lowest possible number of strides) + 2′15″ running at 105% of MAS; RTS: 5’
10	6S of: = Squat (3R at 86% 1RM) + extended bounds (cover 50 m alternating legs by doing the lowest possible number of strides) + 2′ running at 110% of MAS; RTS: 5’
11	Uphill running. Di: 200 m; I: 115% of MAS; Incl: 6%; S: 3; R: 5; RTR: 3′; RTS: 10′
12	Uphill running. Di: 200 m; I: 120% of MAS; Incl: 6%; S: 2; R: 5; RTR: 3′; RTS: 10′

I: intensity; S: sets; R: repetitions; RTS: resting time between sets; RTR: resting time between reps; 1RM: one-repetition maximum; MAS: maximum aerobic speed; Di: distance; Incl: inclination.

**Table 2:** Training methodology that will be used with ETG.

Week	Training Parameters
1	Fartlek training. Du: 50′; I: 117–162 b.p.m.
2	Fartlek training. Du: 55′; I: 117–162 b.p.m.
3	Fartlek training. Du: 60′; I: 117–162 b.p.m.
4	Continuous training; Du: 55′; I: 135–139 b.p.m.
5	Continuous training; Du: 50′; I: 139–144 b.p.m.
6	Continuous training; Du: 45′; I: 144–149 b.p.m.
7	Extensive interval training (long intervals); I: 159–162 b.p.m.; 10S of 3′; RT: 2′
8	Extensive interval training (long intervals); I: 162–165 b.p.m.; 10S of 2′30″; RT: 2′
9	Extensive interval training (medium intervals). I: 165–168 b.p.m.; 14S of 1′30″; RT: 2′
10	Extensive interval training (medium intervals). I: 168–171 b.p.m.; 16S of 1′; RT: 2′
11	Repetition training. I: 180 b.p.m.; 5R of 3′. RT: 8′
12	Competition method. I: 100% of competition running pace; 1R; Di: 3.5 km

Du: duration; I: intensity; R: repetitions; RT: resting time; b.p.m.: beats per minute; Di: distance.

**Table 3:** Between-subjects comparisons of all the variables assessed: main effect of time, main effect of group, and interaction effect.

Variable	Main Effect of Time		Main Effect of Group		Group per Time Interaction Effect	
	F (1–9)	*p*	F (2–18)	*p*	F (2–18)	*p*
BM	0.31	0.591	0.24	0.785	0.55	0.584
BMI	0.30	0.596	0.86	0.440	0.47	0.632
BFP	13.24	0.005 *	0.42	0.662	3.92	0.055
LM	4.35	0.006 *	0.25	0.780	0.92	0.413
CMJ	42.93	<0.001*	7.48	0.004 *	18.62	<0.001 *
1RMsquat	216.38	<0.001 *	1.92	0.175	27.62	<0.001 *
RE12	194.51	<0.001 *	0.127	0.882	23.08	<0.001 *
RE14	85.14	<0.001 *	20.86	<0.001 *	23.95	<0.001 *
VO_2_max	52.59	<0.001 *	0.12	0.891	8,72	0.002 *
AnT	109.84	<0.001 *	1.39	0.275	37.31	<0.001 *

Legend: BM: Body mass; BMI: Body mass index; BFP: Body fat percentage; LM: Lean mass; CMJ: Countermovement jump; 1RM squat: One-repetition maximum squat; AnT: Anaerobic threshold; VO_2_max: Maximum oxygen consumption; RE: Running economy; *p*: Level of statistical significance; F: Variation between sample means/variation within the samples; *: Significant improvement between the pre-test and post-test.

**Table 4:** Results obtained by the three experimental groups in the pre- and post-test in all the variables assessed.

Variable	Group	Pre-Test		Post-Test		Cohen’s *d*	*p*
		$\bar{X}$	SD	$\bar{X}$	SD		
BM	RSSTG	67.11	5.37	67.49	5.32	0.076	0.159
	ETG	65.80	4.38	65.62	4.30	0.037	0.330
	CTG	66.96	4.48	67.10	4.31	0.066	0.172
BMI	RSSTG	21.27	1.87	21.38	1.76	0.066	0.190
	ETG	20.68	1.31	20.63	1.34	0.051	0.645
	CTG	21.47	1.03	21.52	1.08	0.098	0.171
BFP	RSSTG	15.28	1.03	15.04	1.16	0.199	0.152
	ETG	15.09	0.95	14.86	0.87	0.233	0.051
	CTG	15.18	1.07	14.32	1.15	0.721	<0.001 *
LM	RSSTG	56.77	4.58	57.31	4.71	0.115	0.110
	ETG	55.84	3.59	55.81	3.73	0.006	0.108
	CTG	56.73	4.29	57.50	4.21	0.141	0.018 *
CMJ	RSSTG	33.29	1.71	37.07	1.81	2.141	<0.001 *
	ETG	32.86	1.73	32.42	2.33	0.210	0.446
	CTG	33.06	1.41	35.16	1.29	1.551	<0.001 *
1RM squat	RSSTG	83.10	4.51	90.30	4.57	1.571	<0.001 *
	ETG	83.61	2.27	84.71	3.37	0.348	0.168
	CTG	82.70	2.75	86.30	2.66	1.321	<0.001 *
RE12	RSSTG	41.63	3.07	42.41	3.01	0.272	<0.001 *
	ETG	40.49	2.65	42.76	2.75	0.838	<0.001 *
	CTG	41.61	3.08	42.55	2.71	0.783	<0.001 *
RE14	RSSTG	49.44	3.41	50.28	3.17	0.253	<0.001 *
	ETG	48.92	3.13	50.08	3.33	0.346	<0.001 *
	CTG	48.58	3.12	50.18	3.01	0.431	<0.001 *
VO_2_max	RSSTG	60.29	4.18	60.91	4.51	0.138	0.097
	ETG	59.02	3.01	60.91	4.27	0.581	<0.001 *
	CTG	58.67	3.25	60.98	3.36	0.207	<0.001 *
AnT	RSSTG	14.95	0.68	15.05	0.83	0.131	0.172
	ETG	14.90	0.51	16.05	0.83	0.845	<0.001 *
	CTG	14.65	0.58	15.70	0.42	2.749	<0.001 *

Legend: BM: body mass; BMI: body mass index; BFP: body fat percentage; LM: lean mass; CMJ: countermovement jump; 1RM squat: one-repetition maximum squat; AnT: anaerobic threshold; VO_2_max: maximum oxygen consumption; RE: running economy; *p*: level of statistical significance; *: significant improvement between the pre-test and post-test.

## Data Availability

The data presented in this study are available on request from the corresponding author.
